# Peptides before and during the nucleotide world: an origins story emphasizing cooperation between proteins and nucleic acids

**DOI:** 10.1098/rsif.2021.0641

**Published:** 2022-02-09

**Authors:** Stephen D. Fried, Kosuke Fujishima, Mikhail Makarov, Ivan Cherepashuk, Klara Hlouchova

**Affiliations:** ^1^ Department of Chemistry, Johns Hopkins University, Baltimore, MD 21212, USA; ^2^ Department of Biophysics, Johns Hopkins University, Baltimore, MD 21212, USA; ^3^ Earth-Life Science Institute, Tokyo Institute of Technology, Tokyo 1528550, Japan; ^4^ Graduate School of Media and Governance, Keio University, Fujisawa 2520882, Japan; ^5^ Department of Cell Biology, Faculty of Science, Charles University, BIOCEV, Prague 12800, Czech Republic; ^6^ Institute of Organic Chemistry and Biochemistry, Czech Academy of Sciences, Prague 16610, Czech Republic

**Keywords:** origins of life, protein evolution, prebiotic polymers, early peptides

## Abstract

Recent developments in Origins of Life research have focused on substantiating the narrative of an abiotic emergence of nucleic acids from organic molecules of low molecular weight, a paradigm that typically sidelines the roles of peptides. Nevertheless, the simple synthesis of amino acids, the facile nature of their activation and condensation, their ability to recognize metals and cofactors and their remarkable capacity to self-assemble make peptides (and their analogues) favourable candidates for one of the earliest functional polymers. In this mini-review, we explore the ramifications of this hypothesis. Diverse lines of research in molecular biology, bioinformatics, geochemistry, biophysics and astrobiology provide clues about the progression and early evolution of proteins, and lend credence to the idea that early peptides served many central prebiotic roles before they were encodable by a polynucleotide template, in a putative ‘peptide-polynucleotide stage’. For example, early peptides and mini-proteins could have served as catalysts, compartments and structural hubs. In sum, we shed light on the role of early peptides and small proteins *before* and *during* the nucleotide world, in which nascent life fully grasped the potential of primordial proteins, and which has left an imprint on the idiosyncratic properties of extant proteins.

## 1. Introduction

Proteins are the macromolecules responsible for performing the vast majority of biological functions in extant life, and yet their importance during the Origin of Life is often underappreciated or even neglected. The RNA world hypothesis has recently become recentred at discussions of life's origins, an epistemic shift which can be attributed to remarkable developments in the abiotic chemical syntheses of the four canonical nucleosides [1,2] as well as perhaps equally impressive demonstrations of RNA catalysis discovered by directed evolution—in particular, in mediating RNA replication [3–6]. Recent work suggesting potentially prebiotic pathways to deoxynucleosides [7,8] have moved some to refer to this early period as a ‘nucleotide’ world. One of the logical extensions of this corpus of work is that a catalytically active hereditary molecular system could have emerged directly from a system of abiotic molecules with low molecular weight. In this train of thought, proteins' (or peptides’) role in early life's emergence is typically not discussed and is construed as occupying a later phase, potentially after the emergence of translation. Hence, proteins become ‘important’ once they can be encoded and synthesized in accordance with a nucleic acid template. This model has some intuitive appeal because, in extant biochemistry, proteins do not replicate themselves.

We believe that this way of thinking is over-simplistic at best, and likely incorrect. Named by the Swedish biochemist Jöns Jacob Berzelius after the ancient Greek word π*ρ*ώ*τειο*ς (meaning ‘first’), proteins were indeed most likely the earliest biopolymer. The evidence for this comes from many lines of research, including: (i) the ease with which amino acid building blocks can emerge spontaneously through simple and unsupervised gas-phase chemistry [9,10]; (ii) the prevalence of some canonical amino acids (cAAs) in carbonaceous meteorites [11,12]; and (iii) the facile nature of the condensation reaction between amino acids [13] which can be mediated by wet–dry cycles in ‘warm little pond’ terrestrial settings, or under high-pressure high-temperature conditions in hydrothermal vents [14–16], and may even be possible in the interstellar medium as well [17]. These aspects—while perhaps less high-profile than some recent works focusing on nucleoside and nucleic acid chemistry—merit our utmost consideration as we try to build more detailed models about the abiotic-to-biotic transition (table 1). The goal of this review is to discuss aspects of early proteins and their potential roles *prior to* and *during* the nucleotide world, which we refer to as a ‘peptide-like/nucleoside stage’ and a ‘peptide-polynucleotide stage’.

**Table 1.** Prebiotically relevant properties of polypeptides versus polynucleotides.

consideration	section	polypeptides	polynucleotides
abiotic synthesis of building blocks	2	amino acids—trivial and documented at high concentration without human intervention [9,10,18,19]	nucleosides—possible though non-trivial, and requires changes in reaction conditions, and possibly not compatible with Hadean environment [1,2,7,18]
modularity	3	yes—proteins with smaller/alternative alphabets can fold and be functional [20–28]	partial—each base type requires a base-pairing partner [29–31]
abiotic condensation	2	possible through wet–dry cycling, activation with small molecules (e.g. COS), salt-based deliquescence or catalytic peptide ligation [13,32–34]	possible through wet–dry cycling. Though requires phosphorylated monomers and many branching reactions possible given the various nucleophilic moieties on nucleotides [18,35–37]
functional (catalytic) capacity		diverse. Could have supported early metabolism [38,39]	limited primarily to phosphoryl group transfer chemistry (with the important exception of the ribosome, which catalyses aminolysis of esters) [38]
cofactor utilization	4	diverse [40–42]	limited primarily to Mg^2+^ [43–46]
tolerance to backbone impurity		substitution of amides for esters associated with incremental decreases in stability [13,47,48]	base-pairing possible with diverse backbones (peptides, other sugars [49]), though typically tertiary structures not compatible [50]
pH tolerance		high—stable between 3 and 10	low—stable between 5 and 7—due to both backbone cleavage and depurination
tolerance to high Fe^2+^ levels (and other divalent cations)		high [43,51–53]	low—catalyses hydrolysis of phosphodiesters through ‘in-line’ and Fenton mechanisms [45,46]
unassisted refoldability	5	generally, yes. Complex proteins may require chaperones or translation, but simple proteins can fold unassisted [54,55]	generally, no. Rough energy landscapes mean that energy input or active processes necessary to fold to a single structure [54,55]

Numerous other traits of proteins that have not been widely considered make them an ideal proto-biomolecule. A list of many of these properties is given in table 1 and will be discussed in the following sections. One important property of proteins is that their alphabet is reducible as well as extendable (§3). Several lines of evidence suggest that the protein alphabet existed originally in a reduced form [56–58], such as (i) the ease with which certain cAAs (referred to as ‘early’ amino acids) are abiotically synthesized and the preponderance of similar amino acids on meteorites [59], (ii) the records of genetic code and metabolic pathway evolution [18] and (iii) the inferred amino acid composition of ancestral genomes [60,61]. Moreover, proteins with reduced prebiotic alphabets are still capable of folding into globular-like structures [20–24], and performing molecular recognition [62–64], suggesting that nature could take advantage of the structural and functional potential of polypeptides even with many cAAs missing. Nucleic acids are also capable of having their alphabets reduced [65]; however, the chemical logic of base-pairing places more restrictions on addition to and removal from the alphabet, as each nucleobase type is greatly diminished without a ‘well-chosen’ partner with which it preferentially basepairs.

Extant proteins are known to further extend their functional toolbox by using a range of cofactors (§4). While most important in extant oxidoreductases, cofactors could have played many more roles during prebiotic times before the acquisition of the more sophisticated (late) cAAs, and moreover hark back to a time when prebiotic systems chemistry was very diverse, prior to the establishment of canonical components through a central encoding dogma.

**Figure 1.**
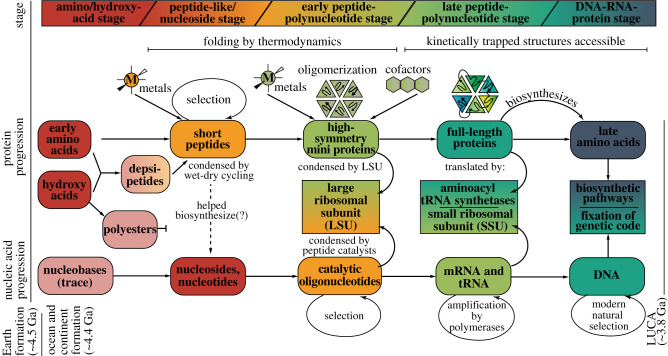
A model for the Origin of Life informed by the early accessibility of various monomeric organic molecules leading to the formation of short peptide-like molecules and emphasizing the various ways in which peptides and nucleic acids coevolved through collaborating at successive stages of sophistication. Section 2 describes these five stages: the amino/hydroxy acid stage, the peptide-like/nucleoside stage, the early peptide-polynucleotide stage, the late peptide-polynucleotide stage and the DNA–RNA–protein stage.

Peptides enjoy one of the simplest and most versatile condensation reactions in organic chemistry, between an amine and a carboxylic acid. The condensation reaction between two amino acids can be mediated through the removal of water without any catalysts [13,34], by activation with a range of prebiotically plausible condensing reagents [66], or potentially catalysed heterogeneously by minerals [19,67]. Thanks to this, a recent network model predicted that iteratively combining products from reactions in seven ‘generations’ from a starting set of six gases (N_2_, CH_4_, NH_3_, H_2_O, H_2_S, HCN) would culminate in 27 different peptides [68]. By contrast, the condensation reaction of nucleotides presents several challenges. First, the selective incorporation of 5′–3′ phosphodiester linkages represents a regioselectivity challenge, given the simultaneous presence of 2′ hydroxyls [35,36]. Second, nucleobases possess numerous nucleophilic functional groups, which must compete with the 5′ and 3′ hydroxyl groups on the sugar as the donor to the phosphate group in condensation. Hence, the creation of linear polymers (as opposed to the combinatorially more facile highly branched structures) poses a statistical challenge [37]. Third, the double negative charge present on terminal monophosphates render them quite unreactive without activation or catalysis [69]. These challenges therefore invite the speculation that condensation of nucleotides was *catalysed* [35], with peptides possibly playing a role. This hypothesis—if true—argues for a ‘recentring’ of prebiotic systems chemistry [39] in which non-encoded peptides played an essential role at the earliest stages [70]. Moreover, it suggests that polynucleotides (and perhaps nucleosides) were themselves the product of early biocatalysis. This scenario provides an alternative to a purely ‘organic chemical’ emergence of polynucleotides and invites a new way of thinking about the origins of life that combines the ‘best’ of what peptides and nucleotides had to offer at distinct stages of emergence (figure 1).

## 2. Protein selection without nucleic acids

In figure 1, we lay out a model for the Origin of Life that integrates a series of emergences in the development of polypeptides and polynucleotides, and emphasizes important ways in which these molecules ‘collaborated’ with each other at different stages.

### 2.1. Amino/hydroxy acid stage

The amino/hydroxy acid stage reflects a very early period in which amino acids and alternatives to amino acids (such as hydroxy acids or dicarboxylic acids) accumulated on the Hadean Earth through Miller–Urey reactions in the atmosphere and by delivery from carbonaceous meteorites [18]. Hydroxy acids are highlighted here along with amino acids as their formation of polyesters or depsipeptides (when both ester and amide linkages form in a mixture of amino and hydroxy acids) under wet–dry cycle conditions has been studied as an appealing prebiotically plausible mechanism for peptide bond formation. Depsipeptides have been shown to be enriched with amino acids over time through a combination of ester–amide bond exchange [13]. In our view, the much higher concentration of these building blocks relative to nucleobases (by *ca* 3–4 orders of magnitude [12,71]) and their more facile synthesis from small gases imply an earliest stage where amino acids dominated the portion of the primordial soup destined to become biotic. In essence, the universe's chemical ‘preference’ for amino acids gave the protein progression (top row of figure 1) a ‘leg up’ on the nucleic acid progression (as illustrated by the staggered colour scheme in figure 1) and influenced the state of each of these molecule types' progressions at the subsequent stages.

### 2.2. Peptide-like/nucleoside stage

In a peptide-like/nucleoside stage, peptides with structural and functional properties expanded by self-templated synthesis without a nucleic acid template. The last few years have witnessed a dizzying number of examples of proteins (oftentimes through short peptide motifs) self-associating in myriads of ways with diverse aggregation numbers and degrees of order. Liquid–liquid phase separated droplets mediated by disordered regions and rigid beta-amyloids represent two ends of this spectrum with respect to order, though remarkably both are often mediated through short peptide-length motifs. These findings bear great significance for the earliest prebiotic stages, when the peptides that were available were probably short and non-globular.

A large body of work has demonstrated the ability of short peptides to self-assemble into a range of morphologies, including fibres, nanotubes, ribbons and vesicles. All of these morphologies are enabled by ‘open’ self-complementarity which allow stable structures to form with simple polymers (*N* as low as 5 [72] or 6 [73]) and high aggregation numbers. A range of examples have demonstrated how such structures could have propagated, in a prion-like manner, through nucleation, growth and fragmentation, in a manner mimicking self-replication [74–76]. Moreover, there is some evidence that such amyloidogenic sequences can self-replicate by employing the amyloid's ‘layered’ structure to spatially organize monomers at an open face, thereby biasing condensation reactions to generate peptides of like sequence [77–79]. Self-assembling peptide sequences, organized into cross-beta structures (or other stable morphologies) would also be expected to be more resistant to hydrolysis than other short peptides. Therefore, under wet–dry cycling conditions, structure-forming peptides could be actively selected for at the expense of non-structure-formers, both at the level of synthesis and at the level of hydrolysis. Also, such hydrolytic conditions would have provided a natural chemical evolutionary pressure to purge hydroxy acid constituents from depsipeptides toward an eventual takeover by peptides [13] (figure 1). Nevertheless, the tolerance of hydroxy acids into peptide-like polymers likely provided a helpful stepping stone for functional polymers to emerge at this early stage [47,48].

Therefore, template-based self-assembly of such peptide pools could lead to a type of ‘imperfect replication’, providing a mechanism to induce amino acids to polymerize into particular sequences over the myriad of alternative possibilities. We note that because of the traditional emphasis paid to the auto-replicative capacities of polynucleotides in the Origin of Life community, more investigation of the self-propagating capabilities of peptides is necessary, though there are a few examples in the literature [66,79,80].

It has been shown that simple amyloids can act as biocatalysts in a number of reactions [66,81–83]. A consideration that has received less attention is that these peptide catalysts may have played important roles in increasing the availability of nucleobases through catalysing elementary chemical reactions, given their paucity in the amino/hydroxy acid stage. Hence, the early emergence of peptides may have supplied the power of biocatalysis to support the synthesis of more challenging building blocks, such as nucleosides and nucleotides. It is also quite plausible that chemical evolution at this stage led to the ‘optimized’ four nucleobase types that we know today, which have been proposed by several others to be products of natural selection [29–31].

During this time period, ribonucleosides and perhaps deoxyribonucleosides also emerged [1,2,7]—though at concentrations significantly lower than those of amino acids, whose polymers already would have had time to undergo considerable selection for specific sequences with favourable properties. This scenario would explain the nucleoside-recognizing capacity of some highly conserved elementary peptide motifs, such as OB-folds and the Walker A motif. Because longer polynucleotides probably required more sophisticated biocatalytic intervention, we refer to this earlier period as the peptide-like/nucleo*side* stage.

### 2.3. Early peptide-polynucleotide stage

In the early peptide-polynucleotide stage**,** longer amino acid sequences arose which can form soluble mini-proteins by taking advantage of homo-oligomerization, metals and organic cofactors to support folding into globular entities. Such mini-proteins differ from the peptides of the earlier stage in that they form ‘closed’ symmetry homo-oligomers, in contrast with smaller peptides that tend to self-assemble into structures with much higher aggregation number through ‘open’ symmetry (point-group versus space-group). These globular mini-proteins in association with metals and cofactors may have supported a primitive metabolism capable of harnessing energy from redox gradients [84] and higher standard free energy substrates (in contrast with the previous stage, where ‘energy’ came primarily from changes in the activity of water inherent in wet–dry cycles). Evidence for this transition can be found in the mutual occurrence of peptide-length sequences in structurally unrelated domains; these elements have variously been referred to as ‘supersecondary structures' [85] or ‘themes’ [86]. It has been previously noted that many of the most elementary protein folds (e.g. TIM barrels, ferredoxins and P-loop NTPases) have an inherent repetitiveness [86–89] which might be traced to short peptide motifs oligomerizing together.

On the other hand, condensing these longer peptide chains required a more efficient catalyst, notwithstanding the fact that homo-oligomeric mini-proteins would not be as amenable to auto-replication as amyloidogenic peptides would. Many paths to ‘replication’ of such mini-proteins are conceivable. It has been proposed that they could be selected for based on their ability to associate with polynucleotides, finding its apotheosis in the emergence of a large ribosomal subunit (LSU). In the LSU, these two molecule types found a symbiosis in which peptides benefitted from a catalyst that could more efficiently condense amino acids, while RNA benefitted from the protective shell afforded by its peptide binders [44,90–95].

At this early stage, nucleotide polymerization may have been carried out by peptide-assisted ribozyme [43,96] or by the mini-protein catalysts themselves, representing another example of symbiosis. Even though several examples of RNA-directed RNA polymerase ribozymes have been reported [3–6], these systems face several criticisms on their claim to prebiotic relevance: (i) the requirement of triphosphorylated building blocks, (ii) the length and topological complexity of the ribozyme, (iii) reliance on divalent cation concentrations of the order of 100 mM and (iv) absence of their existence in the biological record (in contrast with the ribosome, which remains to this day). By contrast, the fact that extant RNA polymerization uses protein-based catalysts and the recent discovery that the core fold of RNA polymerases (the double psi beta barrel) [25] can be reconstituted with a limited amino acid alphabet suggest that RNA may have always been polymerized with a protein-based instrument. Hence, these ideas highlight the importance of RNA–peptide coevolution in enabling RNA polymerization as well as peptide polymerization [97,98].

### 2.4. Late peptide-polynucleotide stage

Without doubt, one of the most important transitions during the prebiotic period was the emergence of a functioning translator in which protein sequences could be specified and encoded from a nucleic acid template. This functionality, which ushered in a ‘late’ peptide-polynucleotide stage, was not a small order: it required the emergence of the ribosomal small subunit (SSU), a set of aminoacyl-tRNA synthetases (aaRSs) to accurately set a genetic code, and a continuous supply of RTP (R = {A, G}; note that the peptidyl transfer reaction, mediated by the LSU, does not require ATP, and in principle could have taken advantage of a range of activated acyl precursors). The advent of translation fully relieved peptides of the need to self-assemble or self-select to perpetuate particular sequences, and at this stage, polynucleotides exclusively take on the role of informational polymer. We shall discuss the evidence for why and how certain extant protein folds became selected prior to their encoding by nucleic acids and why the LSU probably preceded the SSU (see §5).

At this stage, full-length proteins (rather than mini-proteins) can be routinely synthesized, and this obviates the requirement for proteins to fold by oligomerizing small motifs. Polynucleotide segments encoding such motifs stochastically undergo duplication events, resulting in tandem repeats, in which each copy is then free to mutate, resulting in more specialized and less symmetrical proteins. While the parsimony of this type of evolutionary model is appealing, it is always important to point out that convergent evolution cannot be fully ruled out. Specific examples of this mechanism of evolution have been noted in the case of beta propellers [99], the KH domain [100] and Walker-type P-loop NTPases [89,101]. Moreover, the advent of chaperone systems like Hsp60 (GroEL) and Hsp70 (DnaK) further expand the classes of proteins that can be efficiently synthesized [102,103].

### 2.5. DNA–RNA–protein stage

This stage is characterized by the emergence of a DNA-encoded genome, whose building blocks are supplied through ribonucleotide reductases, and which can be replicated in a high-fidelity, highly processive manner by DNA polymerases. Prior to this stage, DNA and RNA could have been interchangeable and perhaps even co-mingled into polynucleotides with both types of monomers [7,8]. Because of the large size and complexity of modern DNA polymerases and ribonucleotide reductases compared to many other types of proteins, we imagine this at the final stage in our protein–nucleic acid coevolution scheme prior to LUCA, as it presupposes a translational machinery capable of synthesizing large multi-domain proteins. This stage also saw the completion of the canonical genetic code and the stable addition of ‘late’ cAAs which had to be biosynthesized metabolically (see §3). We envision the fixation of the amino acid alphabet as a late event in this sequence of events, as amino acids such as Trp, Tyr, Lys and Arg involve numerous enzymes in their biosynthesis and therefore likely required a DNA-based genome to retain the suite of catalysts necessary to prepare them. An important question that is worth reflecting upon (as we [20,24,104] and others [21–23,26–28,105] have shown) is how proteins managed to complete fairly sophisticated functions in the previous two stages without the full complement of amino acids (see below). The hallmark of this stage is the end of ‘chemical evolution’ as Darwinian biological evolution takes root thanks to the intrinsic stability of information encoded in DNA (compared to RNA), and the high (but not perfect) fidelity of polymerases which create the potential for point mutations, insertions and duplications.

## 3. Amino acid alphabets

Despite its wide variety, all life that we know uses the same alphabet of 20 cAAs (and rarely also selenocysteine and pyrrolysine, as the 21st and 22nd) to code for its proteins. There is ample evidence that the protein alphabet emerged via chemical and biological selection from a set of prebiotically available compounds to its current form [56,57,60,106]. Detailed analyses imply that the current set of 20 (as well as its hypothetical subsets) has unique adaptive properties compared with equal sets of random alternatives [107,108]. Within the chemical space, the canonical alphabet represents unusually optimal spectra of size, charge and hydrophobicity when compared with alternative alphabets [109]. These observations have added weight to a long-standing hypothesis that gradual incorporations of individual amino acids would probably steer the fitness landscape in a similar way, producing near identical sets if amino acid alphabets were to evolve on other Earth-like planets [108,110].

At the same time, the last two decades of biological engineering has informed us that many of the cAAs can be removed or substituted and that proteins can be constructed using amino acids beyond the canonical alpha-amino acids [111,112]. This line of research has been inspiring scientists to search the amino acid chemical space and to define similarly optimal ‘xenoalphabets’ [113]. Corresponding ‘xenoproteins’ would represent a great future tool to compare the canonical and alternative alphabets' optimalities with respect to creating polymers with structure and function.

### 3.1. Early versus late amino acids

**Figure 2.**
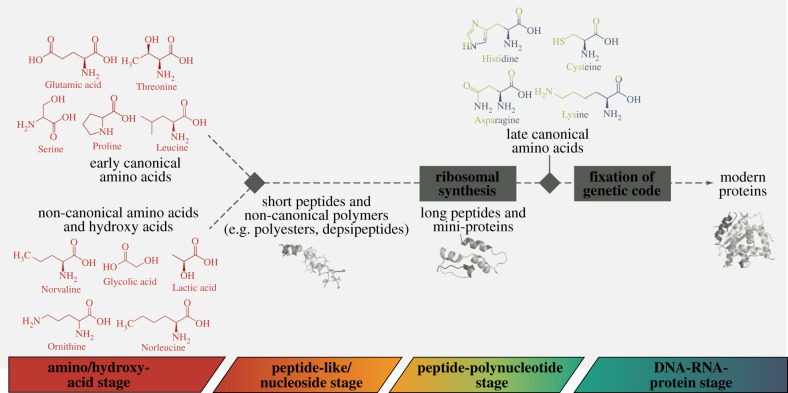
A model for the evolution of the amino acid alphabet.

During the earliest prebiotic stages, the chemical space that was accessible was probably limited to environmentally available compounds. The sources of these prebiotically plausible amino acids were both endogenous (synthesized on Earth in e.g. hydrothermal vents and atmospheric mixtures) and exogenous (delivered from outside the Earth, e.g. by meteorites). More than 80 and 20 different amino acids have been identified in meteorites and atmospheric spark discharge simulations, respectively, with only half of the 20 cAAs highly represented among these [10,114]. Three independent meta-analyses ranked the prebiotic plausibility of amino acids and their additions to genetic code, agreeing on roughly the same 10 ‘early’ amino acids within the current canonical alphabet [18,56,57] (figure 2). These early cAAs are Ala, Asp, Glu, Gly, Ile, Leu, Pro, Ser, Thr and Val. The other half of the modern protein alphabet comprises amino acids with higher synthetic costs, more complex structures and higher reactivity, and were most likely products of catalysts and metabolism [56,58]. Cysteine is not included in the list of early amino acids in the available meta-analyses, but it has been pointed out that the majority of the studies that these were based on did not include sulfur in the atomic mix of the experiments. When H_2_S was later included in the Miller–Urey experiment, possible cysteine degradation products were detected suggesting that cysteine could be produced in primordial synthesis but was unstable and oxidized [10].

### 3.2. The early alphabet (smaller versus messy alphabet)

Preceding templated ribosomal proteosynthesis, early peptides were most probably constructed from a more diverse pool of monomers found in the prebiotic environment (figure 1). As mentioned above, a plethora of non-canonical amino acids (ncAAs) and a broader class of amino acid structures, and even some alternatives to amino acids (such as beta- and gamma-amino acids, hydroxy acids or dicarboxylic acids) have been detected in different prebiotic sources and their possible involvement in building early polymers has been considered by several researchers. Some of the alternative linear aliphatic amino acids (such as alpha-aminobutyric acid, norvaline and norleucine) have been detected in various prebiotic settings in similar amounts as the early cAAs [115–117]. These amino acids have been moreover identified as promiscuous targets of some aminoacyl-tRNA synthetases, suggesting that these ncAAs may have had earlier relevance even in the evolving genetic code [118,119]. Similar debates have been rising about the positively charged amino acids as none of the canonical ones (Lys, Arg and His) have been observed among the early set of the alphabet. At the same time, their ncAA analogues with fewer methylene groups (such as ornithine, 2,4-diaminobutyric acid and 2,3-diaminopropionic acid) appear to be more accessible prebiotically [120]. In today's life, cationic amino acids are indispensable and especially key for interaction with nucleic acids [121]. Their absence among the early alphabet represents a barrier to many hypothesized scenarios about protein evolution and hence an important role played by their ncAA analogues during early stages of the genetic code development has been proposed [101,122–124]. Nevertheless, several examples in the literature now show that folding of acidic proteins can be assisted by metal ions or other cationic species to compensate for the lack of positive charges [20,21].

It has been argued that the beta- and gamma-ncAAs (which have been also identified prebiotically, albeit usually in lower yields when compared with alpha-AAs) and polymers built of similarly prebiotically available compounds (such as the aforementioned hydroxy acids or dicarboxylic acids) would be less prone to form secondary and tertiary protein structures than alpha-AAs [106,110]. At the same time, this does not rule such oligomers out of possible prebiotic relevance [106]. Both helical and beta-sheet-like conformations have been observed in beta-AA polymers [125]. Much attention has been recently devoted to polyesters and depsipeptides that have been shown to form during model prebiotic reactions driven by wet–dry cycles from alpha-hydroxy acids or combinations of alpha-AAs and alpha-hydroxy acids, respectively [13,126]. Although such polymers are less stable than peptides, they nevertheless can form secondary structures [127], and it has been argued that they could have served as an important intermediate during chemical evolution. In conclusion, it seems very probable that short peptides incorporating ncAAs and alternative monomers preceded ribosomal synthesis during the peptide-like/nucleoside stage. During the early peptide-polynucleotide stage, the earliest LSU-synthesized peptides also likely incorporated ncAAs and alternative monomers prior to protein synthesis according to an RNA template [128,129]. Their potential role in shaping protein structure/function remains to be better described by the origins of life and synthetic biology communities. Further evolutionary selection could have produced the canonical genetic code by fixing some early cAAs, purging others, and supplementing the early canonicals with the later structurally and functionally more complex additions.

### 3.3. Selection of the late amino acids

Although several analyses suggest the probable sequence of the late amino acid incorporation into the genetic code, many debates and questions about the order and factors that influenced these events remain open [56,130,131].

To start with, cysteine is regarded as one of the latest additions to the code according to the Trifonov meta-analysis [130]. At the same time, it is one of the most active and unique amino acids involved particularly in Fe–S clusters (such as in ferredoxin, considered one of the earliest protein domains) and conflicting hypotheses have been proposed as to whether these features were indispensable in early evolution. Powner's group made an argument towards indispensability of Cys in early biological processes, while Moosmann *et al*. suggested that Cys-mediated features could be either ignored or replaced in LUCA [33,131]. One of the main arguments for the late emergence of Cys in the AA alphabet was the absence of its plausible prebiotic synthesis pathway, although this has been recently challenged, albeit in near-neutral pH and low-temperature conditions [33]. However, as mentioned above, many of the prebiotic synthesis experiments did not include sulfur in the source material and when they did, possible degradation products of cysteine were detected probably as a result of its oxidation and therefore implying its conceivable prebiotic synthesis [10]. The ease of Cys degradation was also listed as a factor for its later importance in Wong's theory of amino acid alphabet evolution [132]. Interestingly, the Cys biosynthesis pathway was successfully re-engineered using enzymes lacking cysteine residues, providing an important proof-of-concept that a Cys biosynthetic pathway could be supported by proteins lacking this amino acid [133]. As an aside, it is important to point out that ferredoxin's emergence was also discussed in terms of a simple ‘theme’ comprised of early amino acids (initially without cysteine) duplicating and later adding cysteine, conforming to the overall model in figure 1, with the addition of late amino acids representing a relatively late stage during pre-LUCA evolution [134].

Another noteworthy amino acid is histidine, which is the most widely employed catalytic residue in enzymes [135]. It was hypothesized that His might have come from a catalytic nucleotide and could be derived from pre-existing purines [118]. At the same time, Shen *et al*. showed the possibility of non-enzymatic His synthesis, which was later disputed as unrealistic under prebiotic conditions [123,136]. Interestingly, Lazcano *et al*. postulated that the extant biosynthetic pathways of His and purine syntheses have evolved separately, with purine synthesis predating that of His [123]. His is therefore regarded as a late amino acid along with the other positively charged cAAs, despite their high relevance in today's protein alphabet. As described above, it has been argued that their important role was substituted by some of the prebiotically plausible basic ncAAs.

Finally, Met, Trp and Tyr are considered the latest additions to the amino acid alphabet [58,130]. A study by Granold *et al*. [58] suggests that they were incorporated into the genetic code during the great oxidation event as they showed antioxidant properties. At the same time, this event would render the employment of Cys toxic to cells, which posed a challenge that early life had to cope with. Intriguingly, the Granold *et al*. study also suggests that Cys might have been an earlier addition to the AA alphabet than previously estimated. An important final consideration to raise is that some of the late cAAs may have arisen transiently or were present in low concentrations in specific environments at earlier stages; we would argue, however, that their ‘stable’ presence (and certainly their incorporation into a genetic system) would have required metabolism.

### 3.4. Protein consequences of the evolving alphabet

The earliest peptide/protein-like polymers were probably random (statistical) sequences [137,138]. Using modern tools of synthetic biology, several groups have mimicked random sequences from the canonical alphabet or its reduced subsets, in search of their general properties (summarized in Tong *et al*. [139]). In short, random sequences can inherently form secondary structures similar to their occurrence in biological proteins and between 5 and 20% of random peptides of lengths 80–100 amino acids have been reported capable of undergoing compaction/folding [140–143]. Specific functions have been selected from libraries of random or highly randomized sequences implying that the structural and functional propensities of randomly generated peptides are compatible with an early role in evolution [85,144–146]. Importantly, some of these studies have also informed us that proteins constructed from the limited subset of the early canonical alphabet are in fact more soluble, similarly prone to secondary structure formation and perhaps structurally more compact than if built from the full alphabet [20,147–149]. Hence random peptides likely provided a well-spring of potential, from which chemical evolution could act to select individual species with favourable structural, catalytic or compartmentalizing properties.

Complementary top-down approaches, or ‘reverse evolution’, have been used to study the effect of the alphabet reduction on protein structure/function of select protein targets. Most of the earlier studies that reduced the amino acid composition (of small selected proteins such as a beta-trefoil fold, SH3 domain and nucleoside kinase) towards the early cAAs reached an alphabet size of 10 to 13 and/or 80–90% early AA composition [22,23,26,27,105]. These studies reported that folding as well as activity can be preserved in these potentially ancient sequences, although decreases in both structural stabilities and catalytic activities were observed. Longo *et al*. pointed out that reduced protein stability can be improved by a halophilic environment when aromatic core packing interactions are missing in the structure [22,27]. The studies from the Akanuma group argue that the early cAAs are sufficient for folding and stability while the late cAAs were recruited to achieve efficient catalysis [23,28]. By contrast, the work by Longo *et al*. [27] suggests that some of the late cAAs are crucial for the evolution of structural stability. A recent mutation study of a dephospho-CoA kinase where all the aromatic amino acids were substituted resulted in the loss of structural stability, though a transition from a molten globule-like structure to a compact functional fold was observed upon ligand binding [21]. The emerging scenario is that while less stable and less functional mini-proteins can still be constructed in the absence of late cAAs, the deficiencies of these proteins (e.g. lacking positively charged and aromatic amino acids) can be compensated for by high salt concentration, the presence of divalent metal cations or binding to organic cofactors. In agreement, a study by the Tawfik group observed that polyamines and divalent cations can promote folding of highly acidic proteins [21]. We have recently observed that under cell-like conditions, random sequences formed from the 10 early cAAs exhibit similar structure-forming propensity as the full alphabet repertoire despite their very acidic nature. Unlike the full alphabet proteins, they are intrinsically more soluble and exhibit these properties independent of molecular chaperone activities [104]. Importantly, an RNA-binding domain was recently reconstructed using an alphabet of only the 10 early cAAs, uncovering metal cation mediated interaction between the RNA and negatively charged cAAs [20]. Along with a recent reconstruction of an RNA-binding peptide incorporating ornithine as a prebiotically available cationic ncAA, the study by Giacobelli *et al*. provides an important lead to how an early metabolism could function in the absence of late cAAs [20,101]. It is intriguing to speculate that the early preference for acidic AAs over basic AAs (which in turn primarily coordinate metal cations over halogen or chalcogen anions) left a lasting imprint to modern biology in that: (i) virtually all extant proteomes are more acidic than basic [150], (ii) cells maintain metal cations at significantly higher concentrations than elemental anions (the most abundant anion in most cells is in fact glutamate, an early cAA) and (iii) signalling disproportionately uses cations over anions. Therefore, prebiotically plausible ncAAs, metal cations and cofactors have had a lasting impact on extant proteins.

## 4. Evolutionary significance of cofactors

Cofactors are essential components of many of today's proteins. They stabilize certain protein structures and are required for the catalytic activity of many enzymes. Most of the core cofactors are highly conserved across the three domains of life (with some important exceptions among methanogens, e.g. coenzyme M and factor F430), and they would have played an important role in the earliest evolution of peptides, i.e. during the peptide-like/nucleoside and early peptide-polynucleotide stages according to the model presented above (figure 1). From a chemical point of view, cofactors can be divided into two major classes: inorganic cofactors represented by metal ions (§4.1) and organic cofactors (§4.2).

### 4.1. Metal cation cofactors

In modern biology, various metal cations (K, Mg, Ca, V, Cr, Mn, Fe, Co, Ni, Cu, Zn and Mo) are involved in over half of the functionally annotated proteins [40], playing an important role in diverse catalytic functions (especially electron transfer) and maintaining the structural integrity of many protein folds. The overall abundance and accessibility of these metal ions has been affected over the evolution of Earth due to changes in the redox state of the ocean and the atmosphere via geochemical and biological processes [151]. While minerals likely played an important role in prebiotic chemical evolution [152] and also could have been part of metal cofactor-associated protein catalysts [153], in this section, we mainly discuss the contribution of metals in their soluble cationic form.

The estimated time interval for the origin of life on Earth ranges from 4.5 Ga to 3.7 Ga (Hadean to early Archaean), according to evidence of earliest habitability and the biosignature boundary [71]. If abiotic peptides existed and contributed to the origin of life and their early evolution, the accessibility of metal ions presumably shaped the types of proto-metalloenzymes that catalysed the key chemical reactions to sustain the primordial biological system. During the Hadean–Archaean period, trace amounts of oxygen favoured highly soluble Fe^2+^ in reducing environments, whereas the accessibilities of Mo, V and Cr were limited [154]. Mn, Co and Ni were present in the Archaean ocean, presumably in the high nM to μM range, but Cu and Zn were believed to be extremely scarce [151].

A recent reconstruction of ancestral metalloenzymes [155] showed, paradoxically, a universal preference for Mo over Fe^2+^ in nitrogen fixation, indicating that the selection of metal elements for proto-metalloenzyme catalysis might not be solely determined by global geochemical abundance, but also actively a consequence of selection for function [156]. Moreover, the local abundances of metals along with their interacting peptides vary between each niche environment and therefore drive different chemical reactions. For example, high concentrations of the transition metals Zn^2+^ and Mn^2+^ (which were globally rare during the Archaean) could be achieved in a geothermal pond where cooled geothermal fluids and condensed vapours resulting from a volcanic activity can enrich certain metal ions based on their difference in boiling temperature [51]. Such vapour condensed environment is favourable to concentrate K^+^ ion over Na^+^ and thus considered as a plausible environment for protocell evolution to achieve the consistent high K^+^/Na^+^ ratio that we see in almost all modern cells.

The alkaline earth metal ions Mg^2+^ and Ca^2+^ are deeply involved in diverse biological functions. A model considering continental weathering and hydrothermal alteration of seafloor crust estimates that in the Archaean ocean, Mg^2+^ and Ca^2+^ existed at mM concentrations with higher abundances for Ca^2+^ [157]. Overall, these metal ions were very accessible, both globally or locally, in the Hadean–Archaean ocean and terrestrial sites—an observation that is consistent with their importance in supporting the structure and functions of primitive polypeptides and polynucleotides.

Many translation-related proteins such as tRNA synthetase and translation factors require Mg^2+^ (in some cases Zn^2+^ is also needed) for function and in addition use Mn^2+^ for structural integrity [51]. Mg^2+^ plays many different roles in tRNA synthetases such as ATP binding, amino acid activation and the pyrophosphorolysis of the aminoacyl adenylate [52]. We would like to point out that while the prebiotically available concentrations of Mg^2+^ would have been sufficient for binding to peptides and stabilizing RNA tertiary structures, it would not have been high enough for the activities of many ribozyme replicases that have been developed by laboratory evolution. This discrepancy may suggest an earlier dependence of polynucleotides on peptides, which can relieve the requirements for high Mg^2+^ [43].

Based on the MetalPDB [158], we found that the carboxylate moiety of the two acidic amino acids (Asp or Glu) is strongly associated with Mg^2+^ binding through electrostatic interaction, along with several minor examples of coordinating amino acids such as Asn, Gln and Ser. It is notable that Mg^2+^, which was an important cofactor during the earliest stages, is typically coordinated by early amino acids, and not by the late amino acids His and Cys, which are more significant for coordinating Cu and Zn. Hence, this observation supports the view that Asp, Glu, Mg^2+^ and Ca^2+^ represent an ‘early cohort’ of amino acids and metals, while His, Cys, Cu and Zn^2+^ represent a later cohort. Indeed, the carboxylate moiety in minimal metal-binding peptide motif (DXDXD) has been reported to chelate Mg, Mn, Ni and Zn in various modern enzymes and thus has been proposed as one of the earliest metallopeptides [159].

**Figure 3.**
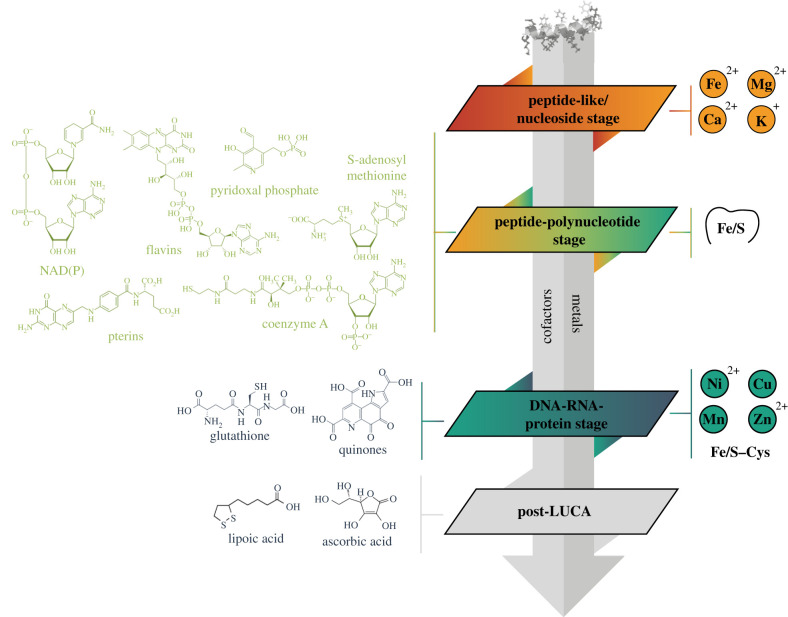
Chronology of the addition of organic cofactors and metal cations at distinct stages.

One of the most studied examples of metal coordination within a protein–RNA complex is the ribosome. Mg^2+^ has long been identified for contributing to the overall maintenance of its structure by serving as a counterion to the phosphate moieties and is also concentrated within the peptidyl transferase centre (PTC) [44]. Importantly from a prebiotic perspective, Mg^2+^ in the ribosome can be substituted with Fe^2+^, a metal that was more abundant in early oceans [53]. A recent study by Rozov and co-workers unveiled the positions of K^+^ ions within the ribosome, indicating the involvement of K^+^ in mediating rRNA–rRNA and rProtein–rRNA interactions, as well as in the PTC by increasing the stability of rRNA and tRNA [160]. It is interesting to note that K^+^ ions were found in pockets formed by the negative ends of the dipoles of carbonyl oxygen atoms from the polypeptide backbone. Similarly, clefts consisting of backbone N–H groups form so-called ‘nest’ structures [161] which are also important in binding various anionic groups such as phosphates, sulfates, carbonates and iron–sulfur (Fe/S) centres [162]. A good example is the Gly-rich P-loop Walker-A motif that binds ATP or GTP. Because of this simple binding mode with very little side chain involvement, nest motifs of oriented N–H groups and carbonyls within peptide loops might be considered as one of the earliest functional protein motifs [163]. It is appealing to hypothesize that such nest motifs could have occurred in the early Cys-less ferredoxins, employing backbone N–H groups to coordinate Fe/S centre, perhaps in addition to organic sulfides such as methanethiols; indeed even modern ferredoxins show a distinct bias to orient N–H dipoles toward their iron–sulfur centres (figure 3).

The other prebiotically abundant alkaline earth element is Ca^2+^, which is widely used in intracellular signalling in eukaryotes, and also frequently stabilizes proteins in thermophiles [164]. Ca^2+^ exhibits a high affinity for carboxylates and rapid binding kinetics (100-fold faster than Mg^2+^) making it a useful metal cofactor [165]. The amino acid chelators for Ca^2+^ are similar to those of Mg^2+^; namely, predominantly Asp and Glu, followed by several other supporting amino acids (Ser, Thr, Asn and Gly). Unlike Mg^2+^, cellular Ca^2+^ ion is maintained at extremely low concentrations (10^−7^ M) to prevent Ca^2+^ from precipitating peptides, inorganic phosphates and phosphate-bearing biomolecules [166]. The tendency for Ca^2+^ to precipitate phosphate and polynucleotides could possibly explain biology's preference for Mg^2+^ and why active mechanisms are used to pump it into specific membrane-bound compartments in modern cells. From an early prebiotic context, however, when ion-impermeable compartments and active efflux mechanisms were likely not available, these observations raise the question as to whether early stages of life sought environmental niches where other anions could have depleted Ca^2+^ from solution.

Finally, considering the emergence and evolution of metabolism, transition metals are essential for the redox chemistry to provide key precursors of biomass from simple inorganic compounds including the essential atoms C, H, O, N, S and P. In view of the global abundance on early Earth and the versatility of chemical reactions, Fe and Fe-bearing minerals clearly stand out due to their non-enzymatic reactions resembling those of ancient cofactors [167] and core metabolic pathways [168,169]. For example, warm acidic Fe^2+^-rich water can promote a reaction network recapitulating most of the biological TCA and glyoxylate cycle intermediates [170]. Partly electro-reduced FeS (Fe/S-Fe^0^) is able to catalyse reductive amination leading to the formation of several amino acids from α-ketoacids and ammonia under alkaline condition [171]. These results indicate the versatility and importance of iron-promoted protometabolism, which was eventually taken over by enzymes that harbour metal centres and organic cofactors due to their improved efficacies and specificities [172].

### 4.2. Organic cofactors

Some organic cofactors are considered evolutionary ancient molecules of prebiotic origin, while others are probably the inventions of early biochemical metabolism [41]. In most cases, they likely originated independently of proteins [173] and the binding of cofactors to primitive polypeptides appears to have been a critical step in protein evolution. The early cofactors might have facilitated protein formation as catalysts (to build amino acids or peptide segments), as molecular chaperones (to facilitate protein folding), and/or as selectors (because of the important function of early cofactors) [173].

Based on the available studies, cofactors can be divided approximately into three categories based on their evolutionary age: (i) ancient cofactors (associated with the peptide-polynucleotide stage) include those that could have been synthesized under prebiotic conditions and therefore existed before the establishment of protometabolism; (ii) early cofactors (associated with the DNA–RNA–protein stage) include chemical moieties that most likely appeared only after the emergence of the first proto-cells but were present in the last common universal ancestor (LUCA); and (iii) late cofactors were developed after the divergence of three domains of life from LUCA (figure 3).

The most ancient cofactors are thought to include nucleotide-derived cofactors (NAD(P), FMN, FAD, coenzyme A, S-adenosylmethionine, pterins and pyridoxal phosphate). Many of these are composed of ribonucleoside or nucleotide units (NAD(P), coenzyme A, S-adenosylmethionine) or they are biosynthetically derived from nucleotides (FMN, FAD and tetrahydrofolic acid). Several cofactors (NAD(P), FAD and coenzyme A) contain AMP as a structural element which is not involved in catalysis but rather serves as a ‘handle’ for binding to enzymes [174]. Nucleotide-containing cofactors together with inorganic cofactors represent two main groups of cofactors that are assumed to be of prebiotic origin and played the primary role in protein evolution [42]. While metal ions and minerals that resemble metal ion clusters found in the modern proteins (such as Fe/S clusters) should have been widespread in the primordial Earth environment, nucleotide-containing cofactors' provenance in the peptide-polynucleotide stage provides a direct testament to how these two types of molecule types coevolved intimately during early chemical evolution [175–177]. Nucleotide-derived cofactors may have helped facilitate the jump from peptides to mini-proteins (figure 1). Ji *et al*. [173] noted that domains associated with binding nucleotide-derived cofactors are among the most ancient as based on the diverse range of folds associated with binding these cofactors as a core function. For instance, there are 35 folds associated with binding ATP, 27 for NAD(P), 21 for FAD, 16 for FMN, 15 for GTP, 14 for CoA and 13 for SAM. Collectively, this argues that the earliest globular domains were probably selected for their ability to bind cofactors, an activity that was particularly salient in the absence of late amino acids. This observation is in agreement with the current understanding of evolutionary history of protein folds according to which the P-loop NTP hydrolase fold, the adenine nucleotide alpha hydrolase fold (both using ATP), the flavodoxin-like fold (using NADH, NADPH and FADH_2_), the SAM-dependent methyltransferases fold (using SAM) and the NAD(P)-binding Rossmann fold are among the most ancient protein structures [178–181]. The highly ancestral nature of domains which bind to nucleotide-derived cofactors serves as indirect evidence that such cofactors may have helped stabilize ancient versions of these globular proteins (via ‘induced folding’), which were likely divided up into more (and shorter) polypeptide chains, and lacked the greater stability that could be imparted by incorporation of late AAs and longer chain lengths.

## 5. Refoldability

**Figure 4.**
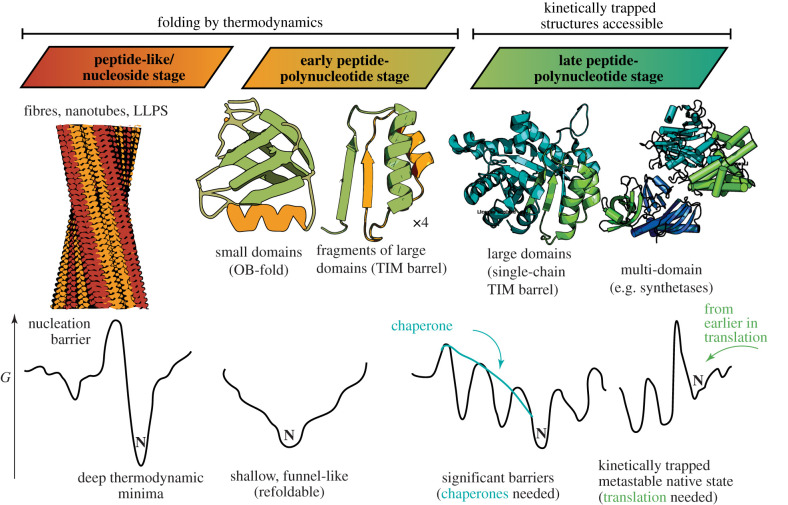
Chronology of peptide and protein topologies available at different stages from the perspective of foldability.

Sophisticated macromolecules need to be able to fold into well-defined globular structures and maintain those conformations to perform their functions. This capability likely emerged during the early peptide-polynucleotide stage when peptide chains long enough to create a hydrophobic core arose (though it should be noted that due to symmetry and self-assembly, they did not need to be as long as some of today's globular domains, which emerged in the late peptide-polynucleotide stage). That proteins can fold into a single (or small number of) well-defined conformation(s) can be explained by the fact that stable folded structures require hydrophobic residues to form a tightly compacted core, but they have idiosyncratic shapes, and are connected together through a continuous chain that can only bend in specific ways. Satisfying these requirements simultaneously gives protein folding a puzzle-like quality that results in relatively few solutions. Stated another way, the free energy landscape that describes globular polypeptides has a funnel-like architecture with a single minimum (or a small number of minima), ensuring that the native state reflects a thermodynamically stable state, and that there are many possible paths to get there from an arbitrary unfolded state (figure 4).

### 5.1. The significance of refoldability during the early peptide-polynucleotide stage

Funnel-like energy landscape topologies endow polypeptides with the important feature of reversible refoldability. A consequence of this trait is that when a small protein suffers some shock (in temperature, pressure, pH or other condition) that causes it to unfold, it can return to its native structure without any external assistance upon returning to native conditions. The observation that many simple proteins are refoldable implies two features about a protein's energy landscape: first that the native state is indeed the global minimum, and second that it is accessible on a given timescale (i.e. barriers and traps en route to the native state are not too high or deep).

Refoldability was probably an essential feature for early proteins during the early peptide-polynucleotide stages. Because of the absence of a dedicated proteostasis machinery, the only protein quality control system that was available was thermodynamics. A recent work has suggested that LUCA had only one major chaperone, namely Hsp60 (GroEL), as even the essential quasi-universal Hsp70 (DnaK) may have only appeared after the archaea–bacteria divergence [102]. However, GroEL requires a continuous supply of ATP, which necessitates metabolism and hence was probably not available until the *late* peptide-polynucleotide stage. While GroEL would have unlocked protein folds that are harder to reach due to intervening traps and barriers, the biomacromolecules of the early peptide-polynucleotide stage must have had native states that were straightforward to access, unassisted, by following a free energy gradient.

Because refoldability represented an important feature for the earliest proteins, folds that display this canonical biophysical attribute were likely present earlier during the origin of life. A recent study by To *et al*. [182] interrogated the refoldability of the *E. coli* proteome and found that two-thirds out of approximately 1200 proteins were reversibly refoldable on a biologically relevant timescale of 2 h. This group was enriched with monomeric proteins (75% refoldable), single-domain proteins (70% refoldable), small proteins (less than 20 kDa, 80% refoldable) and proteins without any annotated domains (and hence, more likely to be disordered, 87% refoldable).

It was also found that some of the folds that are believed [180,181] to be the most ancient (e.g. OB-folds, 3-helix bundles, ferredoxin-like domains, flavodoxin-like domains, SH3 domains and SAM-dependent methyltransferase domains) were predominantly refoldable (greater than 80%).

The TIM barrel fold is an interesting case study, because it is also often cited as being one of the most primordial fold types, though the study by To *et al*. found it not to be among the more highly refoldable fold types (65% refoldable). This is consistent with the observation that TIM barrels disproportionately require the assistance of GroEL [183]. The relatively larger size of TIM barrels compared to other elementary globular domains, and their eightfold pseudo-symmetry suggest that in the early peptide-polypeptide stage, TIM barrels' antecedents were able to prevail, in the form of a shorter beta-alpha motif that self-assembled into barrels, but that their concatenation into the single-chain TIM barrels that we know today had to wait until a later stage when chaperones (or translation) became available. This same hypothesis probably also holds for the P-loop NTPase fold, also frequently noted as one of the most abundant and ancient protein folds [184]: it probably started as a simple beta-loop-alpha motif (the so-called Walker-A motif), and its expansion and diversification were driven by the availability of chaperones and translation [89].

One of the unexpected findings in To and co-workers’ study was the finding that virtually all the ribosomal large subunit proteins were refoldable in a complex mixture under prebiotically plausible conditions. On the other hand, many small subunit proteins as well as translation factors were found *not* to be reversibly refoldable. This finding furthers the notion that a large subunit functioning as a ribozyme peptidyl transferase (a ‘proto-PTC’) evolved earlier than and independently of the coordinated decoding of tRNAs by a functional small subunit (figure 1) [90,185].

### 5.2. Chaperones and translation give rise to a late peptide-polynucleotide stage ‘explosion’

The advent of chaperones and translation, which we assign to the late peptide-polynucleotide stage, resulted in the elaboration and diversification of larger harder-to-fold domains, as well as multi-domain proteins. Chaperones play a critical role of burning energy to re-extend proteins that are trapped in an intermediate misfolded state, thereby allowing them a fresh chance to fold, in a mechanism referred to as iterative annealing [186,187]. This function was important for the larger domains like TIM barrels and P-loop NTPases, smoothing the transition from self-assembly of smaller motifs to longer chain lengths that emerged through genetic duplication [188].

Translation had major consequences for the types of proteins that could be easily created because it enables proteins to fold co-translationally [189,190]. Co-translational folding facilitates access to kinetically trapped (metastable) native states because it can ‘seed’ proteins in one region of their energy landscape at an early chain length and then retain them there if synthesis proceeds faster than the egress rate out of that region. It is also generally important for multi-domain protein folding to decouple the folding of individual domains, which would otherwise be prone to generate improper inter-domain contacts [191]. Translation also allows proteins to join together into more diverse complexes by enabling the coordination of protomer folding and subunit assembly. This allows ‘obligate complexes’ to be routinely synthesized. By contrast, the simpler oligomers that were accessible during the early peptide-polynucleotide stage probably needed to be able to reversibly assemble from independently stable protomer units. At this later stage, protein assembly no longer needs to be dictated exclusively by thermodynamics, and kinetically trapped protein assemblies become accessible (figure 1).

To and co-workers found that virtually all the *E. coli* aminoacyl-tRNA synthetases (aaRSs) are nonrefoldable. On one level, this finding is not surprising, given that these enzymes are in all cases multi-domain proteins, and many use more specialized fold types (e.g. anticodon-binding domains, the class II synthetase fold and the HUP domain). Nevertheless, this finding argues for an intriguing point: just as aaRSs are essential for protein translation from a nucleic acid template, aaRSs themselves also necessitate translation (or chaperones) to properly fold. Moreover, the emergence of aaRSs is a prerequisite for the small subunit of the ribosome to perform its core function in tRNA decoding. In other words, long viewed as among the most ancient protein folds, aaRSs may actually be relatively new in comparison to smaller domains or those which can be split into smaller repetitive themes. To summarize, evidence from refoldability argues that the synthetases, the small subunit and translation all bear hallmarks of a later stage of development, and it is likely that the three emerged together because of their mutual interdependence. In our view, these developments defined the late peptide-polynucleotide stage, and with them, the relaxing of the requirement that proteins' native states be easily locatable.

### 5.3. Regarding the refoldability of RNA

RNA is often described as having a ‘rougher’ energy landscape than protein with more near-degenerate minima [192,193]. This character can be attributed to the dominant role of base-pairing, which has an additive quality, and the fact that in RNA, secondary structure is largely decoupled from tertiary structure [55]. As a consequence, many possible conformations with the same (or similar) number of Watson–Crick base pairs are roughly degenerate. This is a major contrast with protein folding, which is characterized by a highly cooperative hydrophobic collapse, and wherein secondary structures are relatively unstable outside the context of a tertiary structure. With these features, single mutations can result in total destabilization of a folded form [194,195].

From a computational perspective, the contrast makes the protein folding problem a more formidable puzzle. But from an Origin of Life perspective, it means simple proteins have a useful trait in their propensity to occupy a single (or small number of) native state(s) that can be reversibly relocated. The intrinsic structural heterogeneity encoded in RNA's energy landscape can be overcome biologically through co-transcriptional folding [196,197], RNA chaperones [198] or suppressed *in vitro* through careful (but arbitrary) annealing schedules or serial dialyses. However, processive RNA polymerases probably only emerged during the late peptide-polynucleotide stage. In the remarkable case of the ribosomal large subunit, which appears to be intrinsically reversibly refoldable, the rRNA refolding process is very likely chaperoned by extensive RNA–protein interactions, wherein rProteins with well-defined tertiary structures induce rRNA to choose specific base-pairing patterns over alternatives [198]. In the other particular case of tRNA (also of ancient provenance, and easily refoldable), refoldability is possible because of high stability and simple topology (i.e. base pairs form across adjacent regions that are separated by short loops).

On the other hand, for intricate ribozymes with topologies more complex than tRNA and fewer protein interactors than ribosomes, inherent refoldability is far from guaranteed. It should give us pause that no ribozyme (aside from the ribosome) is universally distributed or confidently traceable back to LUCA [38,199]. Indeed it has been pointed out that ‘There is no conclusive evidence that intron self-splicing and ribozyme-mediated RNA processing are truly primordial activities’ [199]. We note that ‘strong’ RNA world hypotheses that led to the assertion that LUCA was a protoeukaryote (because eukaryotes alone habour the majority of extant catalytic RNA) are inconsistent with current models for the root of the tree of life [200,201]. Finally, it should also give us pause that the remarkable ribozymes discovered in recent decades through directed evolution have all themselves been birthed from sophisticated and processive RNA polymerases, providing the luxury of co-transcriptional folding that was likely not available until at least the late peptide-polynucleotide stage. More research is necessary to elucidate RNA refoldability, as it remains an understudied area. On balance though, there is preliminary evidence to suggest that complex RNAs ‘leaned on’ peptides and proteins to help tame their rough energy landscapes' proclivity toward structural heterogeneity. Hence, evidence from refoldability argues for another important way in which ancient RNA and proteins needed to cooperate to support key functions during the emergence of life, with the primordial trait of high intrinsic refoldability more generally associated with proteins.

## 6. Conclusion and future outlook

In this mini-review, we have sought to bring together evidence from molecular biology, bioinformatics, geochemistry and biophysics to provide insight into the emergence of proteins during the early stages of the origins of life, prior to LUCA. We advocate for this period to be separated into five ‘stages’ (figure 1): (i) the amino/hydroxy acid stage, (ii) the peptide-like/nucleoside stage, (iii) the early peptide-polynucleotide stage, (iv) the late peptide-polynucleotide stage and (v) the DNA–RNA–protein stage. Through this classification, we seek to highlight the ways in which the antecedents of today's proteins and nucleic acids cooperated and were interdependent on each other at distinct stages of emergence.

Proteins are sometimes viewed as being a later development during the Origin of Life, on account of the fact that they cannot self-replicate in modern biology, and are synthesized in accordance with an RNA template by translation. However, proteins have a number of qualities that make them ideal for supporting early transitions toward biological complexity during the origin of life, including: (i) the abundance of their components from abiotic terrestrial and extraterrestrial sources, (ii) the relative facility of their condensation, (iii) their self-assembly properties into complex morphologies, (iv) their catalytic versatility, (v) the reducibility of their alphabet, (vi) their propensity for intrinsic refoldability and (vii) their capacity to interact with a range of cofactors. Each of these factors played important roles at distinct prebiotic stages. High abiotic abundance of amino acids played an extremely important formative role (during the amino/hydroxy acid stage). Condensation of amides and self-assembly of short peptides (as well as depsipeptides) into large structures were particularly relevant during the peptide-like/nucleoside stage and could have afforded nature some of its earliest molecular scaffolds, compartments and catalysts. The availability of early biocatalysts could have accelerated the availability of other building blocks whose synthesis is more challenging, such as nucleosides and lipids.

Spontaneous folding and refoldability was paramount during an early peptide-polynucleotide stage when quality control mechanisms to maintain biomacromolecules' conformations was not yet available, while meanwhile ancient globular protein folds, composed of smaller self-assembling constituents composed of a smaller palette of amino acids, appeared. These folds had a strong propensity to bind nucleotide-containing cofactors, which expanded catalytic versatility and provided additional stability. Close interactions between such proteins and polynucleotides likely chaperoned polynucleotide folding, while also providing a means for proteins to propagate through association with the more easily replicating polynucleotides. This interdependence resulted in the ribosomal large subunit.

The invention of translation enabled larger proteins to appear that are harder to fold and relieved proteins of any need to propagate themselves. At the same time, protein-based polymerases allowed complex RNA topologies to appear that did not rely on protein binders to help tame their rougher energy landscapes. In our chronology, all of these developments occurred prior to the advent of late amino acids, the fixation of the genetic code and the establishment of DNA-based genomes.

The model we present for the Origins of Life is not without its limitations. The potential of peptides to self-propagate without a nucleic absent template is greatly understudied, and more examples of this behaviour are needed to support the peptide-like/nucleoside stage. Proteomics technologies are still outstripped by nucleic acid sequencing technologies, though are consistently improving, and may help shed much needed light on chemical evolution of peptides. While there are examples of catalytic amyloids, evidence of their catalytic utility being directed toward the synthesis of other prebiotically relevant molecules is lacking and would represent an important discovery. We still have many questions surrounding how specific mini-protein sequences could have been maintained before being directly encoded by replicating genetic material. With the current renaissance underway in Origins of Life research, we are optimistic these currently mysterious aspects can be addressed by future experiments.

At the same time, we hope in this mini-review to motivate a deeper acceptance about the implications of peptide–polynucleotide coevolution. These biomolecule types did not appear in isolation, and this fact should be more reflected in our experiments. For instance, research on prebiotic polynucleotides should consider peptide ‘cofactors’ rather than use unrealistically high divalent cation concentrations [43]. Research trying to resurrect early proteins composed of fewer amino acids should be more inclusive of nucleotide-based cofactors. And prebiotic chemistry should more strongly consider how selectivity could be afforded by simple catalysts, rather than by high-performance liquid chromatography. In general, models that try to assert the preeminence of one biomolecule type over others during the Origin of Life will probably prove incorrect in the long run. Like people, when different types of biomolecules work together, amazing things can happen.

## Data accessibility

This article has no additional data.
